# Inhibitory effects of SEL201 in acute myeloid leukemia

**DOI:** 10.18632/oncotarget.27388

**Published:** 2019-12-24

**Authors:** Ewa M. Kosciuczuk, Aroop K. Kar, Gavin T. Blyth, Mariafausta Fischietti, Sameem Abedin, Alain A. Mina, Rebekah Siliezar, Tomasz Rzymski, Krzysztof Brzozka, Elizabeth A. Eklund, Elspeth M. Beauchamp, Frank Eckerdt, Diana Saleiro, Leonidas C. Platanias

**Affiliations:** ^1^ Robert H. Lurie Comprehensive Cancer Center of Northwestern University, Chicago, Illinois, USA; ^2^ Division of Hematology-Oncology, Department of Medicine, Feinberg School of Medicine, Northwestern University, Chicago, Illinois, USA; ^3^ Department of Medicine, Jesse Brown Veterans Affairs Medical Center, Chicago, Illinois, USA; ^4^ Division of Hematology/Oncology/Stem Cell Transplantation, Department of Pediatrics, Ann and Robert H. Lurie Children's Hospital of Chicago, Chicago, Illinois, USA; ^5^ Division of Hematology and Oncology Department of Medicine Medical College of Wisconsin, Milwaukee, Wisconsin, USA; ^6^ Ryvu Therapeutics, Kraków, Poland; ^7^ Department of Neurological Surgery, Feinberg School of Medicine, Northwestern University, Chicago, Illinois, USA

**Keywords:** acute myeloid leukemia, MNK, eIF4E, kinase inhibitor, SEL201

## Abstract

MAPK interacting kinase (MNK), a downstream effector of mitogen-activated protein kinase (MAPK) pathways, activates eukaryotic translation initiation factor 4E (eIF4E) and plays a key role in the mRNA translation of mitogenic and antiapoptotic genes in acute myeloid leukemia (AML) cells. We examined the antileukemic properties of a novel MNK inhibitor, SEL201. Our studies provide evidence that SEL201 suppresses eIF4E phosphorylation on Ser209 in AML cell lines and in primary patient-derived AML cells. Such effects lead to growth inhibitory effects and leukemic cell apoptosis, as well as suppression of leukemic progenitor colony formation. Combination of SEL201 with 5’-azacytidine or rapamycin results in synergistic inhibition of AML cell growth. Collectively, these results suggest that SEL201 has significant antileukemic activity and further underscore the relevance of the MNK pathway in leukemogenesis.

## INTRODUCTION

Acute myeloid leukemia (AML) is an aggressive hematological malignancy with relatively limited therapeutic options [1]. Abnormal activation of multiple signalling pathways, including mitogen-activated protein kinase (MAPK) pathways, is key in the pathogenesis and pathophysiology of AML. These pathways regulate different cellular processes including leukemic cell proliferation and survival in response to a variety of signals [2–5]. MAPK-interacting kinases 1 and 2 (MNK1/2) are downstream effectors of MAPK pathways and regulate multiple cellular processes through phosphorylation/activation of the eukaryotic translation initiation factor 4E (eIF4E) [6–9], a key component of the translation-initiation complex [10, 11]. MNK1/2 phosphorylation of eIF4E at serine 209 triggers increased mRNA translation of mitogenic mRNAs that promote proliferation, cell cycle progression, and pro-survival processes [12–15]. eIF4E has been found to be overexpressed in a wide variety of human malignancies [16–20] and eIF4E phosphorylation on serine 209 is strongly associated with its transforming capacity [21, 13]. However, neither MNK activity, nor the phosphorylation of eIF4E appear to be essential for normal development [12, 22, 23]. Given that MNK-mediated eIF4E phosphorylation strongly contributes to tumorigenesis, lymphomagenesis, and tumor metastasis while being dispensable for development, pharmacological MNK1/2 inhibition may represent an attractive strategy for the treatment of leukemias [24, 21, 12].

Recently, a new ATP-competitive inhibitor of MNK1/2, SEL201, has been reported to selectively inhibit MNK1 and MNK2 activity, with a half maximal inhibitory concentration (IC_50_) of 10.8 nM and 5.4 nM, respectively, in *in vitro* kinase assays [25]. Importantly, SEL201 was shown to be orally bioavailable, safe and well tolerated in mice [26]. Moreover, SEL201 showed potent antitumor and anti-metastatic effects using KIT-mutant melanoma and breast cancer in *in vitro* and *in vivo* models [25, 26]. In the current study, we examined the antileukemic properties of SEL201 using AML models. We provide evidence that SEL201 suppresses eIF4E phosphorylation on Ser209 in AML cells and such effects appear to result in enhanced cellular apoptosis, and growth inhibitory responses. Notably, combination of SEL201 with 5′-azacytidine and rapamycin resulted in synergistic anti-leukemic effects *in vitro*.

## RESULTS

**Figure 1:**
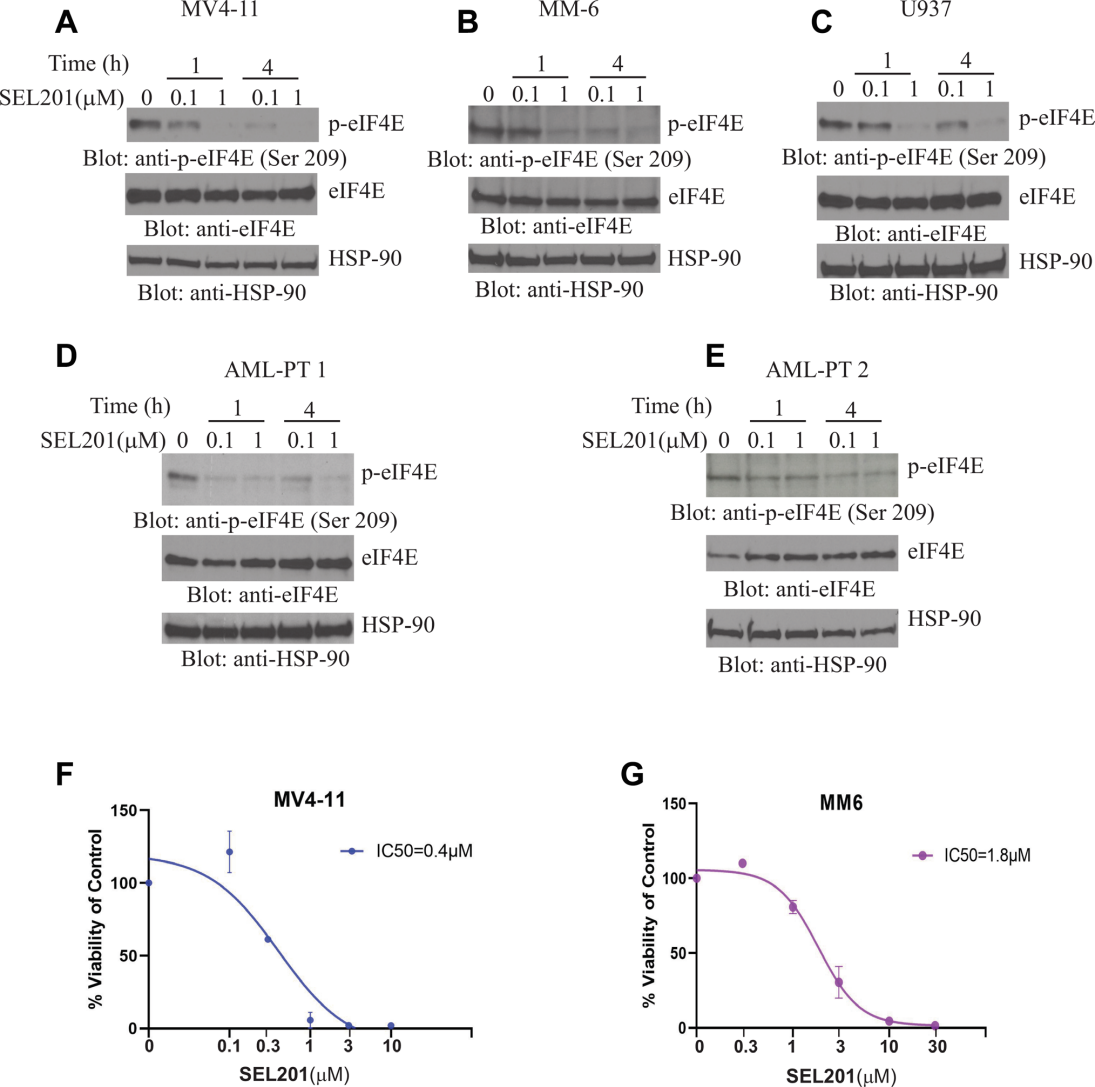
SEL201 suppresses phosphorylation of eIF4E and inhibits cell proliferation in AML. (**A**) MV4-11, (**B**) MM6, (**C**) U937 cells or (**D**–**E**) AML patient-derived cells were incubated with SEL201 for 1 hour and 4 hours at final concentrations of either 0.1 or 1μM. Equal amount of total cell lysates were resolved by SDS-PAGE. Blots were probed with the indicated antibodies. (**F**) MV4-11, (**G**) MM6 cells were plated in 96 well plates and treated with increasing concentrations of SEL201 for 7 days. Viability was assessed using WST-1 assay. Data are expressed as a percentage of control (DMSO-treated) cells. Shown are the means ± SE of 3 independent experiments, each done in triplicate, and IC_50_ values are shown for each cell line.

**Figure 2:**
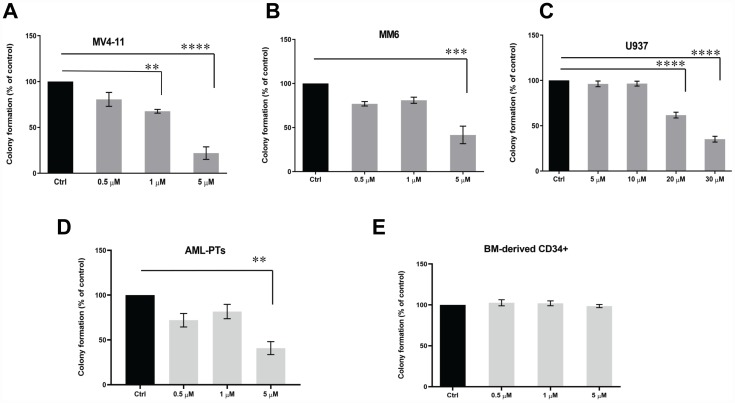
SEL201 exhibits suppressive effects on AML leukemic progenitors, but not on normal hematopoietic progenitors. (**A**) MV4-11, (**B**) MM6 and (**C**) U937 cells were plated in a methylcellulose culture assay system with increasing concentrations of SEL201, as indicated. Data are expressed as percentage of colony formation of control (vehicle-treated) cells, and shown are the means ± SE of four independent experiments for MV4-11 and U937 and three independent experiments for MM6. (**D**) The inhibitory effects of SEL201 on primary leukemic precursors from AML patients were assessed in clonogenic assays in methylcellulose. Data are expressed as percentage of colony formation of control (vehicle-treated) cells. Shown are the means ± SE from four independent experiments, using cells from four different patients with AML. (**E**) Normal human bone marrow-derived CD34^+^ cells were plated in clonogenic assays in methylcellulose with increasing concentrations of SEL201, and myeloid (CFU-GM) progenitor colony formation was assessed. Data are expressed as percentage of colony formation of control (vehicle-treated) cells and represent means ± SE of three independent experiments. One-way ANOVA analysis followed by Tukey’s test was used to evaluate statistically significant differences: ^**^ *p* < 0.01, ^***^ *p* < 0.001, ^****^ *p* < 0.0001.

In initial studies, we examined the effects of SEL201 on eIF4E phosphorylation in AML cells. Treatment of the MV4-11, MM6 or U937 cell lines with SEL201 reduced phosphorylation of eIF4E on Ser209 in a dose and time-dependent manner (Figure 1A–1C). Similar results were obtained when the effects of SEL201 on patient-derived primary AML cells were determined (Figure 1D and 1E). To define the functional relevance of inhibition of eIF4E phosphorylation by SEL201, we next performed cellular viability assays using AML cells. SEL201 treatment suppressed the cellular viability of MV4-11 and MM6 cells with IC_50_ values of 0.4 μM and 1.8 μM, respectively (Figure 1F and 1G). Additionally, we evaluated the anti-leukemic effects of SEL201 on primitive leukemic progenitors in clonogenic assays in methylcellulose. SEL201 treatment resulted in significant inhibition of colony formation (CFU-L) derived from different leukemic lines, as well as in primary leukemic precursors from AML patients (Figure 2A–2D). On the other hand, SEL201 showed no suppressive effects on normal bone marrow (BM) derived CD34^+^ cells in myeloid colony formation (CFU-GM) assays (Figure 2E).

**Figure 3:**
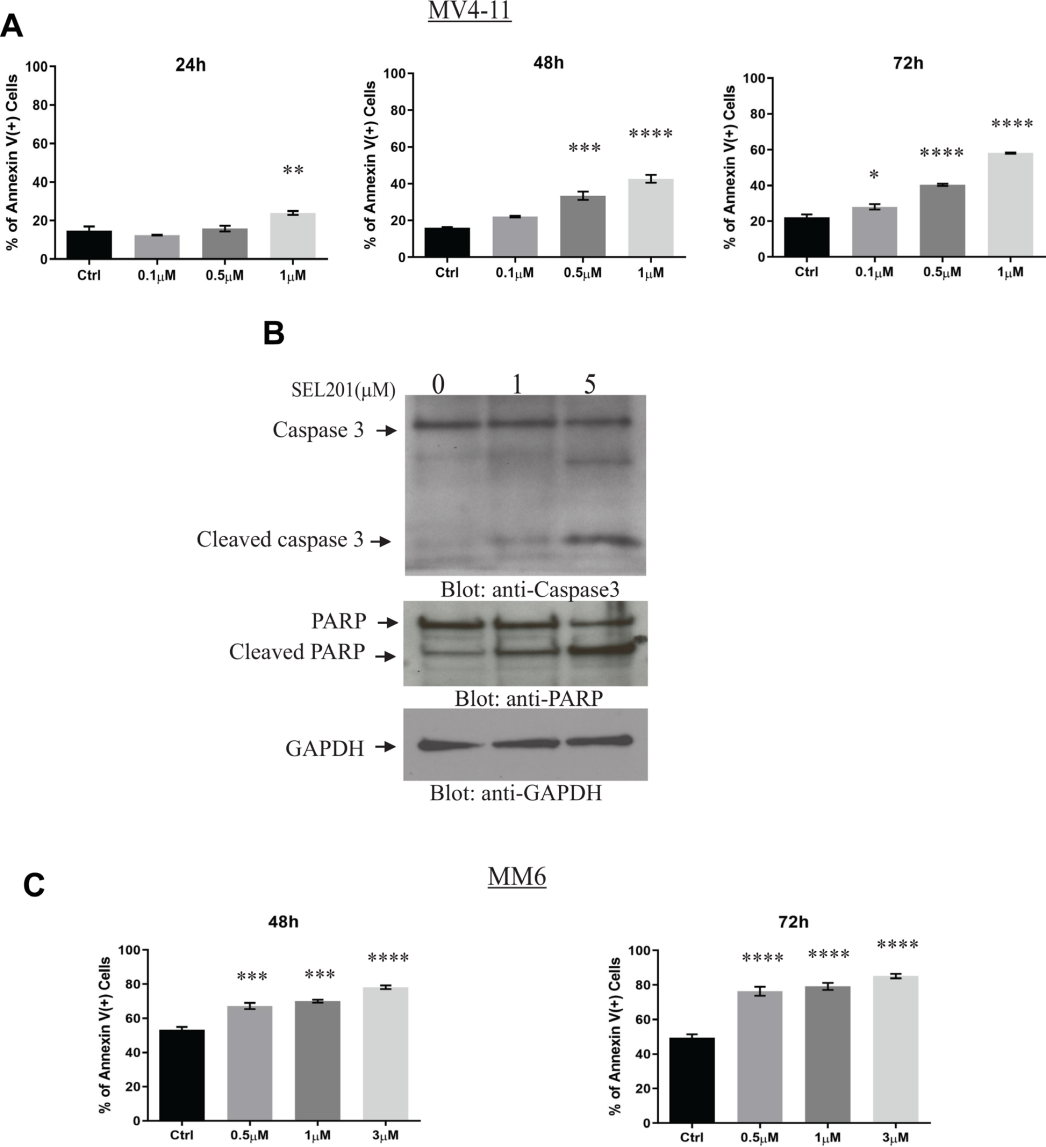
SEL201 induces apoptosis of AML cells. (**A**) MV4-11 cells were treated with SEL201 at the indicated doses for 24, 48 and 72 hours. The percentage of apoptosis was determined by flow cytometry using Anexin V/DAPI staining. Shown are the means ± SE of three independent experiments. (**B**) MV4-11 cells were treated with vehicle or SEL201 at the indicated doses for 24 hours. Whole cell lysates were resolved by SDS-PAGE and immunobloted with the indicated antibodies. (**C**) MM6 cells were treated with SEL201 at the indicated doses for 48 hours and 72 hours. The percentage of apoptosis was determined using Annexin V/DAPI staining followed by flow cytometry analyses. One-way ANOVA analysis followed by Tukey’s test was used to evaluate statistically significant differences between treatments: ^*^ *p* < 0.05, ^**^ *p* < 0.01, ^***^ *p* < 0.001, ^****^ *p* < 0.0001.

In addition to blocking tumor cell viability and proliferation, induction of programmed cell death (apoptosis) is an important effect of many antitumor agents [27]. We examined the pro-apoptotic functions of SEL201 in MV4-11 and MM6 cells using flow cytometry analysis. SEL201 treatment significantly increased the fraction of Annexin-V positive cells in a dose and time-dependent manner, compared to vehicle-treated cells (Figure 3). To further corroborate the induction of apoptosis by SEL201 in AML cells, we assessed the cleavage/activation of the apoptotic markers PARP and caspase 3 by immunoblotting. Treatment of MV4-11 cells with SEL201 resulted in cleavage of both caspase 3 and PARP, consistent with induction of apoptosis (Figure 3B).

**Figure 4:**
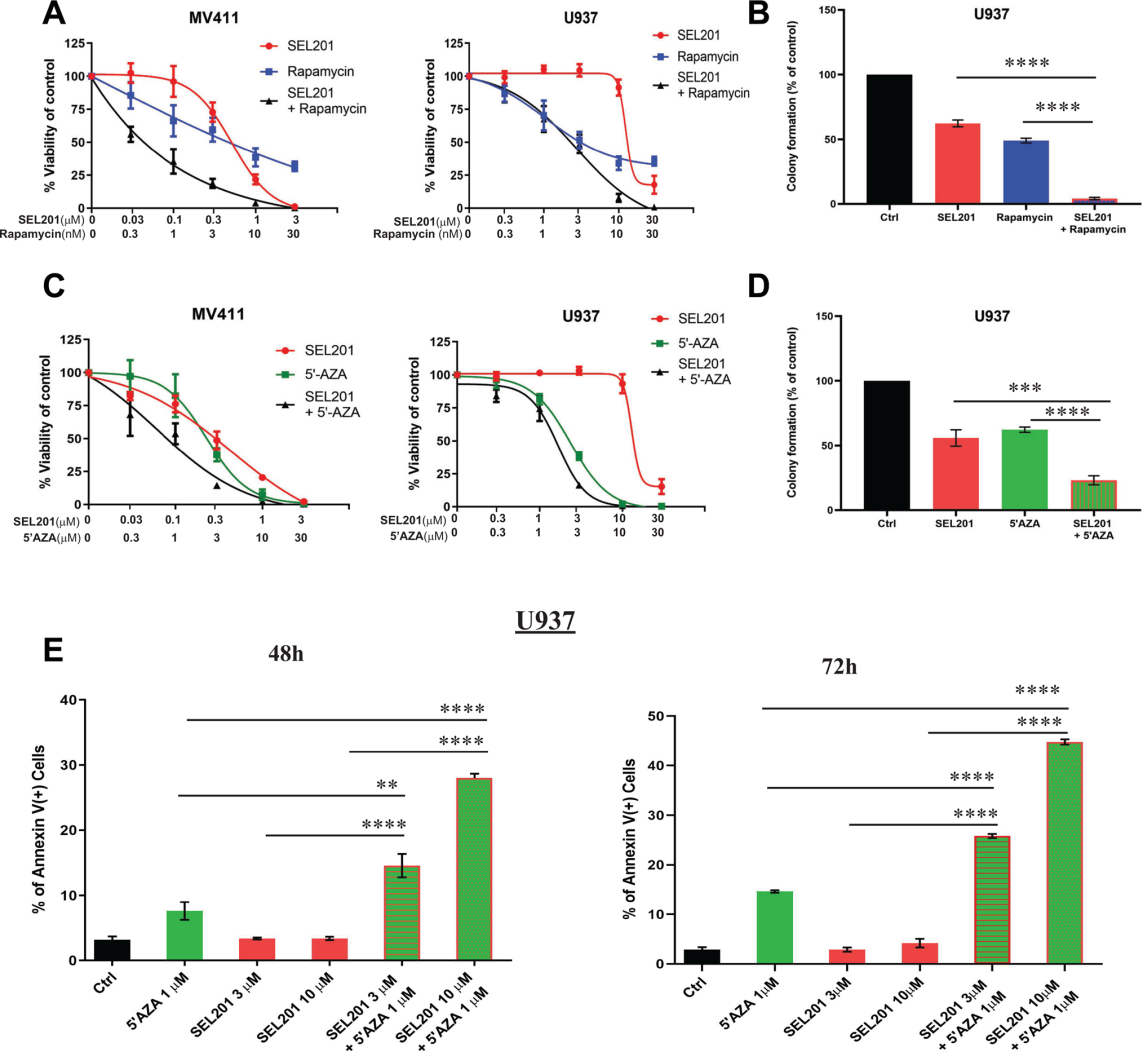
Enhanced antileukemic effects of MNK inhibition combined with rapamycin or 5'-azacytidine. (**A**) MV4-11 and U937 cells were plated in 96 well plates and treated with SEL201 and/or rapamycin for 4 days. Viability was assessed using WST-1 assay. Data are expressed as percentage of vehicle-treated cells (control). Shown are the means ± SE of five independent experiments for MV4-11 and four for U937. (**B**) U937 cells were plated in methylcellulose culture assay system in the presence of SEL201 and rapamycin alone or in combination, as indicated. Data are expressed as percentage of colony formation of control (vehicle-treated) cells, and shown are the means ± SE of four independent experiments. (**C**) MV4-11and U937 cells were plated in 96 well plates and treated with SEL201 and 5′-azacytidine alone and in combination, as indicated, for 4 days. Viability was assessed using a WST-1 assay. Data are expressed as a percentage of vehicle-treated cells (control). Shown are the means ± SE of four independent experiments. (**D**) U937 cells were plated in methylcellulose culture assay system in the presence of SEL201 and 5′-azacytidine alone or in combination, as indicated. Data are expressed as percentage of colony formation of control (vehicle-treated) cells, and represent means ± SE of four independent experiments. (**E**) U937 cells were treated with SEL201 or 5′-azacytidine alone or in combination for 48 hours and 72 hours, as indicated. The percentage of apoptosis was determined using Annexin V/DAPI staining followed by flow cytometry analyses. Shown are means ± SE of three independent experiments. One-way ANOVA analysis followed by Tukey’s test was used to evaluate statistically significant differences between treatments: ^**^ *p* < 0.01, ^***^ *p* < 0.001, ^****^ *p* < 0.0001.

Many patients with AML either do not respond to therapy or often relapse and develop resistance mechanisms to currently used therapies [28, 29], underscoring the need for the development of new treatments for AML patients. Targeting the mTOR and MNK-eIF4E pathways may provide important new opportunities for new cancer therapeutic approaches [30–33]. We evaluated the antitumor combinatorial effect of SEL201 with rapamycin that inhibits the activation of mammalian target of rapamycin complex 1 (mTORC1) [34]. Combination of SEL201 with rapamycin treatment resulted in synergistic inhibition of cell viability of MV4-11 cells (CI = 0.20) and U937 cells (CI = 0.35) (Figure 4A). In addition, the combination of SEL201 with rapamycin significantly enhanced the suppressive effects on leukemic progenitor colony formation (CFU-L) from U937 cells in clonogenic assays in methylcellulose (Figure 4B).

In subsequent experiments we examined whether MNK1/2 inhibition by SEL201 could enhance the antileukemic properties of 5′-azacytidine, a hypomethylating agent with major clinical activity in AML and myelodysplastic syndromes (MDS) [35, 36]. The combination of 5′-azacytidine with SEL201 showed a synergistic effect with a CI value of 0.60 for U937 cells, and an additive effect with a CI value of 1.0 for MV4-11 cells (Figure 4C). Additionally, the combination of SEL201 with 5′-azacytidine significantly enhanced inhibition of colony formation (CFU-L) of U937 cells in clonogenic assays in methylcellulose (Figure 4D). To evaluate whether the potent and synergistic antileukemic effects of combinatorial treatment of SEL201 with 5′-azacytidine correlates with induction of apoptosis, we measured the induction of cellular apoptosis in U937 cells by flow cytometry analysis. We observed a significant increase in Anexin V-positive staining when U937 cells were treated with SEL201 in combination with 5′-azacytidine, as compared to each drug treatment alone (Figure 4E).

## DISCUSSION

MAPK pathways have been previously shown to be constitutively activated in malignant hematopoietic cells and control malignant cell proliferation, via downstream effectors, including activation of MNKs [37, 38]. When activated, MNKs phosphorylate eIF4E, which drives mRNA translation of pro-tumorigenic proteins, such as BCL-2, survivin, cyclin D1 and c-myc [11, 39]. Previous work has shown that MNK1/2 inhibition might block the self-renewal capacity of leukemic cells, without affecting normal progenitor functions [40]. Additionally, eIF4E has been found to be overexpressed in M4/M5 subtypes of AML [41, 42]. Given its involvement in leukemia progression and dispensable role in normal hematopoiesis [40], eIF4E is an attractive therapeutic target in AML. Specific and direct targeting of eIF4E by anti-sense oligonucleotides has previously been used as an eIF4E targeting approach [43]. However, although originally promising, this approach reduced eIF4E expression, but did not substantially improve clinical responses in patients with advanced solid tumors, including colorectal cancer [44, 45]. Given that phosphorylation of eIF4E is crucial for its oncogenic activity, targeting MNK activity might constitute a better strategy for the treatment of malignancies. In fact, several studies have shown that the oncogenic activity of eIF4E can be targeted by inhibiting MNK1/2-induced phosphorylation of eIF4E at serine 209 in different malignancies, including AML, which leads to potent anti-tumor responses *in vitro* and *in vivo* [30, 46–48]. Thus, the selective targeting of this pathway, alone or in combination with other therapies, could be a promising therapy for the treatment of AML.

In the current study, we examined the anti-leukemic effects of SEL201, a novel, orally bioavailable, well tolerated and safe *in vivo*, small-molecule inhibitor of MNK1/2 activity [25, 26]. SEL201 has been recently developed and has been reported to inhibit melanoma clonogenicity, cell migration, and metastasis formation [25], and to induce cell cycle arrest, inhibit proliferation and block invasion and metastasis of breast ductal carcinoma *in situ* [26]. Importantly, SEL201 does not appear to affect proliferation of normal, non-malignant melanocytes [25]. Our studies demonstrate that SEL201 inhibits eIF4E phosphorylation in AML cell lines and primary AML cells, consistent with a direct effect through inhibition of MNK kinase activity. In previous studies blocking eIF4E phosphorylation was shown to suppress mRNA translation of oncogenic genes, leading to inhibition of cancer cells proliferation [26, 46, 47]. In our study, we show that SEL201 suppresses cellular proliferation, viability and clonogenicity in AML. Notably, SEL201 was also found to not suppress the clonogenic capability of normal bone marrow-derived CD34^+^ progenitor cells. Previous studies have shown that eIF4E activation promotes tumorigenesis by inducing expression of anti-apoptotic proteins, such as MCL-1 and survivin [12, 39, 49]. Consistent with this, we found here that inhibition of eIF4E phosphorylation by SEL201 correlates with induction of apoptosis in AML cells.

Cytarabine, an antimetabolic agent currently used for the treatment of AML, was previously shown to induce phosphorylation of eIF4E on serine 209, which could constitute a potential anti-cancer resistance mechanism activated during administration of this therapy [31]. Consistently, cercosporamide, an anti-fungal agent with MNK inhibitory effects, was shown to enhance the antileukemic effects of cytarabine in AML *in vitro* and *in vivo* models [30]. Additionally, ribavirin, an anti-viral guanosine analogue, identified as a direct eIF4E inhibitor, was shown to enhance the therapeutic effects of cytarabine in a clinical trial involving AML patients [41]. Further, drug inhibition of mTORC1 pathways was also shown to trigger phosphorylation of eIF4E via activation of a negative feedback prosurvival pathway [30, 31]. Thus, dual inhibition of mTORC1 and MNK-eIF4E pathways might represent an important strategy for the treatment of several types of cancer [30, 31, 33, 50, 51]. Here, we show that combination of SEL201 with the mTORC1 inhibitor rapamycin exhibits potent antileukemic properties in AML cells lines and primary AML cells. Additionally, we also determined the effects of SEL201 with 5′-azacytidine. Azacitidine (5′-azacytidine) is a clinically approved hypomethylating agent that inhibits DNA methylation of tumor suppressor genes, enhancing their transcription, and has shown significant clinical benefits in patients with myeloid malignancies [35, 51, 52]. Our data show that combination of SEL201 with 5′-azacytidine results in synergistic inhibition of leukemic cell viability and induction of apoptosis of U937, *TP53* mutated, cells. In another study, ribavirin treatment was shown to increase 5′-azacytidine-induced suppression of colony formation of primary AML specimens [53]. In conclusion, our study establishes that SEL201 has antileukemic properties against AML progenitor cells, and further supports the concept of MNK pathways as therapeutic targets in AML.

## MATERIALS AND METHODS

### Cells and reagents

U937 cells were grown in RPMI 1640 medium with 10% fetal bovine serum (FBS) and antibiotics. MV4-11 cells were cultured in IMDM medium with 10% FBS and antibiotics. MM6 cells were grown in RPMI 1640 medium supplemented with 10% FBS, 10 μg/ml human insulin, 1 mM sodium pyruvate, 1mM nonessential amino acids, and antibiotics. Peripheral blood or bone marrow from patients with AML were collected after obtaining informed consent as approved by the institutional review board of Northwestern University. Mononuclear cells were isolated following Histopaque density gradient separation (Sigma-Aldrich). For immunoblotting analysis the mononuclear cells were cultured overnight in IMDM medium supplemented with 20% FBS prior to drug treatment. SEL201 was provided by Selvita S. A. (Ryvu Therapeutics S. A.). 5′-azacytidine and rapamycin were purchased from Sigma-Aldrich (St. Louis, MO, USA).

### Cell lysis and immunoblotting

For immunoblotting experiments, cells were treated with either vehicle-DMSO (control) or SEL201 at indicated doses and time points. Cells were lysed in lysis buffer (50 mM Hepes pH 7.3, 150 mM NaCl, 1.5 mM MgCl_2_, 1 mM EDTA pH 8.0, 100 μM NaF, 100 μM Na_4_P_2_O_7_, 0.5% Triton X-100, and 10% glycerol) supplemented with protease and phosphatase inhibitors. Equal amounts of total cell lysates were resolved by SDS-PAGE and processed for immunoblotting essentially as in our previous studies [54, 55]. Antibodies against phospho-eIF4E (Ser209) (#9741), cleaved Caspase-3 (#9661) Caspase 3 (#9662), PARP (#9542), were from Cell Signaling. Antibodies against eIF4E (sc-9976), HSP-90 (sc-7947) were from Santa Cruz Biotechnology, and anti-GAPDH (#374) antibody was purchased from Millipore.

### Cell viability assays

Cell viability assays were performed as previously described using WST-1 Reagent (Sigma-Aldrich) [46, 56]. For combination therapy, the effect of drug interaction on cell viability was measured by calculating the combination index (CI) using CompuSyn. The CI values were calculated as previously described [56].

### Clonogenic leukemic progenitor assays in methylcellulose

This assay was performed as described in previous studies [46]. Peripheral blood was collected from patients with AML after obtained informed consent approved by the Northwestern University Institutional Review Board. To assess the effect of SEL201 on leukemic progenitor colony formation (CFU-L), mononuclear cells were plated in methylcellulose in the presence of vehicle-DMSO (control) or increasing doses of SEL201 and the indicated agents. Human normal bone marrow CD34^+^ cells (ATCC) were used to assess CFU-GM colony formation in clonogenic assays in methylcellulose (Stemcell Technologies) in the presence or absence of SEL201 at the indicated doses.

### Analysis of apoptosis by flow cytometry

MV4-11 and MM6 cells were treated with either vehicle-DMSO (control) or SEL201 at the indicated times and doses. U937 were treated with vehicle-DMSO (control) or SEL201 and/or 5′-azacytidine for 48 and 72 hours. Samples were processed and analyzed as previously described [46, 56].

### Statistical analysis

All statistical analyses were performed using GraphPad Prism 6.0 software as described previously [46, 56].

## Abbreviations

MAPK: mitogen-activated protein kinase
MNK: MAPK-interacting kinases
AML: acute myeloid leukemia
eIF4EL: eukaryotic translation initiation factor 4E
PARP: poly ADP ribose polymerase
mTORC1: mammalian target of rapamycin complex 1
MDS: myelodysplastic syndromes
